# Self-assembled Collagen-Fibrin Hydrogel Reinforces Tissue Engineered Adventitia Vessels Seeded with Human Fibroblasts

**DOI:** 10.1038/s41598-018-21681-7

**Published:** 2018-02-19

**Authors:** Bijal Patel, Zhengfan Xu, Cameron B. Pinnock, Loay S. Kabbani, Mai T. Lam

**Affiliations:** 1 Department of Biomedical Engineering, Wayne State University, Detroit, MI USA; 2 Department of Vascular Surgery, Henry Ford Health System, Detroit, MI USA; 3 Cardiovascular Research Institute, Wayne State University, Detroit, MI USA

## Abstract

Efforts for tissue engineering vascular grafts focuses on the tunica media and intima, although the tunica adventitia serves as the primary structural support for blood vessels. In surgery, during endarterectomies, surgeons can strip the vessel, leaving the adventitia as the main strength layer to close the vessel. Here, we adapted our recently developed technique of forming vascular tissue rings then stacking the rings into a tubular structure, to accommodate human fibroblasts to create adventitia vessels in 8 days. Collagen production and fibril cross-linking was augmented with TGF-β and ascorbic acid, significantly increasing tensile strength to 57.8 ± 3.07 kPa (p = 0.008). Collagen type I gel was added to the base fibrin hydrogel to further increase strength. Groups were: Fibrin only; 0.7 mg/ml COL; 1.7 mg/ml COL; and 2.2 mg/ml COL. The 0.7 mg/ml collagen rings resulted in the highest tensile strength at 77.0 ± 18.1 kPa (p = 0.015). Culture periods of 1–2 weeks resulted in an increase in extracellular matrix deposition and significantly higher failure strength but not ultimate tensile strength. Histological analysis showed the 0.7 mg/ml COL group had significantly more, mature collagen. Thus, a hydrogel of 0.7 mg/ml collagen in fibrin was ideal for creating and strengthening engineered adventitia vessels.

## Introduction

Tissue engineering blood vessels is an important endeavor for clinical care due to the limited availability of autologous replacement vessels. Although the media and intima layers have received much attention, the adventitia has been given much less consideration despite its crucial role. The adventitia’s main cell type the fibroblast^1^ and its dense collagen I network function as a structural support system by acting as a protective sheath to prevent rupture from increased pressure during blood flow^2^. In surgery, during endarterectomies, surgeons can strip the vessel of all the intima, and most of the media, leaving the adventitia as the main strength layer to hold suture and close the vessel^3^. It is well known that the collagen content in the vessel wall is the main strength of the blood vessels^4^.

In engineered vessels, a variety of biomaterials have been used. Most commonly, polymers have been employed, such as poly(ε-caprolactone) (PCL); poly(lactic acid) (PLA); poly(lactic-co-glycolic acid) (PLGA); poly(ester urethane)urea (PEUU) in various configurations (e.g. conduit/tubular, electrospun, microchannels)^5–9^. Fibrin gels have also been utilized, more so as a base scaffolding material^5,10^. These materials have been primarily used in engineering the media and/or intima layers. Thus, materials selection optimization for engineering of the adventitia has largely not been investigated.

There have been a limited number of engineered vascular grafts utilizing fibroblast cells. L’Heureux *et al*. created autologous human dermal fibroblast cell sheets and wrapped them around a mandrel to create a tube structure^11^. However, to strengthen their adventitia vessel, a 10 week maturation period under perfusion flow in a bioreactor was required. Tranquillo *et al*., conducted an *in vivo* study with their tissue engineered graft made of ovine dermal fibroblasts^12^. Cells were seeded in a tubular mold and cultured for 2 weeks and then placed in a bioreactor for an additional 3 weeks. The common prevailing issue complicating translation is the amount of time taken to strengthen adventitia-based grafts.

In materials selection for engineering the adventitia, it is ideal to choose natural materials which will minimize the risk of chronic inflammation, thrombosis, rejection, and infection. Enhancing vessel strength through material means is achieved by improving cell-to-cell interactions, and extracellular matrix component content and organization. By selecting the optimal material, time to manufacture adventitia vessels can be reduced by circumventing the need for pre-conditioning time. Our lab recently developed a unique technology to create scalable engineered vascular tissue emulating the media layer^13,14^. In our method, termed the “Ring Stacking Method” (RSM), a ring of vascular tissue is created around a center post. The tissue rings are subsequently stacked into tubular structures, creating an engineered vessel. To create the adventitia, we modified the RSM to facilitate formation of human dermal fibroblasts rings and stacks. Base parameters such as cell seeding number, plate size and post dimensions were re-optimized to accommodate fibroblast cell size and growth rates. We found that the fibroblast rings improved strength with supplementation with collagen production stimulating factors. Ascorbic acid has been known to allow formation of a stable extracellular matrix by stimulating fibroblasts, specifically due to increased collagen I production^15–17^. Transforming growth factor- beta (TGF-β) has also been shown to increase collagen production in fibroblast cultures^18,19^. The addition of ascorbic acid and TGF-β, separately and combined, to the fibroblast ring cultures improved ring strength. Although growth factor stimulation is a common tool used in the tissue engineering domain, we discovered that the growth factor stimulation approach for inducing collagen production in fibroblast cells was not alone sufficient to induce adequate collagen to create a robust engineered adventitia. We hypothesized that the addition of type I collagen in a fibrin matrix would increase the strength of engineered adventitia rings and enhance collagen deposition compared to growth factor stimulation alone. Hence, we investigated the efficacy of collagen gel to complement collagen production stimulation effects to significantly increase adventitia ring strength. Differing amounts of collagen gel were tested to determine the optimal quantity necessary for increased tensile strength. Collagen gel was effective in strengthening the rings, and allowed us to achieve significantly higher strength in the engineered adventitia rings and vessels. Amounts were defined by the volume percentage of collagen gel to the total hydrogel. In trialing Fibrin only; 0.7 mg/ml COL; 1.7 mg/ml COL; and 2.2 mg/ml COL gel hydrogels, the 0.7 mg/ml collagen gel rings were the most robust, consistently formed, strongest rings with the highest collagen content and most mature collagen.

## Methods

### Cell culture

Human dermal fibroblasts (HuDF) (PCS-201-012, ATCC, Manassas, VA) were used to create the engineered adventitia. The growth media (GM) consisted of 89% Dulbecco’s Modified Eagle Medium (DMEM) high glucose solution, 10% fetal bovine serum, and 1% antibiotic/antimycotic. Differentiation media (DM) consisted of 98% 231 media, 1% FBS, and 1% antibiotic-antimycotic. The media was changed every 3–4 days during culture. Cells were expanded in 150 mm petri dishes in an incubator at 37 °C and 5% CO_2_ for 7 days, which was the time period needed for the cells to reach approximately 90% confluency. Cells within passage numbers of p5 to p15 were used to maintain healthy morphology.

### Assembly of ring formation plates

The plate assembly method was modified from our previously established methods^13,14^. The base plate used was a 60 mm petri dish. Posts for tissue ring formation were cut out of a block of poly(dimethylsiloxane) (PDMS) polymer (1064291, Dow Corning, Midland, MI) using a 6 mm diameter biopsy punch. The bottom of each of the 60 mm plates were coated with 4 mL of PDMS. The plates were allowed to cure for 48 h at room temperature, after which the 6 mm PDMS posts were centrally adhered to the PDMS coated plate using additional PDMS. The custom-made plates were sterilized with a 30 min soak in 70% ethanol followed by 30 min of UV sterilization under the bio-hood.

### Base hydrogel formulation

Fibrin hydrogel was used in the original ring formation protocol as a provisional matrix to support the cell monolayer as it aggregates towards the central post^13,14^. The fibrin hydrogel component was made using hydrogel media, thrombin and fibrinogen. Hydrogel media consisted of 88.5% 231 media; 0.1% of recombinant human insulin (rH-insulin, 100–11, PeproTech, Rocky Hill, NJ), recombinant human fibroblast growth factor (rH-FGF, 100–18, PeproTech), recombinant human epidermal growth factor (rH-EGF, 100–15, PeproTech), transforming growth factor-beta (TGF-β, 100–21, PeproTech), and ascorbic acid (A8960, Sigma Aldrich, St. Louis, MO); 5% of fetal bovine serum and L-glutamine; and 1% antibiotic-antimycotic. To form the fibrin gel, human type 1 plasma fibrinogen (ICN15112205, Fisher Scientific, Hampton, NH) at a concentration of 20 mg/mL and bovine plasma thrombin (7592, BioVision, Milpitas, CA) at a concentration of 100 U/mL were used in a 4:1 ratio, respectively.

### Adventitia ring formation

Expanded fibroblasts in culture were trypsinized and prepared for seeding. Cell seeding for the rings was tested for seeding in growth medium and differentiation medium to determine the ideal seeding medium for forming the adventitia rings. The cell pellet were either re-suspended in differentiation media or fibroblast growth media depending on their experimental parameter. Each plate was seeded with 4 mL of media either consisting of 5 × 10^5^ cells in GM or 4 × 10^6^ cells in DM. More cells were seeded in DM as compared to GM to account for the slower cell proliferation rate in DM. Fibrin gel was prepared as mentioned above and added to the plate. Media was changed every 2 days. For plates with cells initially seeded with GM, on days 2 and 4 GM was used, then on day 6 and on the media was switched to DM. By day 7, the cells + hydrogel aggregate fully self-organized into rings. Rings were then stacked into vessels on day 8.

### Collagen production stimulation

To strengthen our all-cellular adventitia rings, methods for stimulating collagen production were explored. Ascorbic acid (AA) and TGF-β were chosen as effective collagen production stimulating agents, and different regimens of adding both factors separately and together were investigated. Each ring was supplemented with factors of 100 ng of TGF-β and/or 150 µg of ascorbic acid. Collagen stimulation regimens tested consisted of stimulation on day 1 of seeding followed by three more stimulations until complete ring formation on day 7. The five cytokine stimulation groups were: 1) AA only; 2) TGF-β only; 3) AA administered during seeding and on day 2, then switched to TGF-β for days 4 and 6; 4) TGF-β administered during seeding and on day 2, then switched to AA for days 4 and 6; and 5) TGF-β+ AA administered during seeding and until ring formation on day 7. One day was allowed for the ring to stabilize, enabling the rings for use on day 8.

### Collagen gel effects

In addition to exploring collagen production stimulation in order to increase ring tensile strength, adding pure collagen in the form of collagen gel was also investigated. The collagen gel mixture was prepared using human collagen type I gel (VitroCol, Advanced Biomatrix, Carlsbad, CA) per manufacturer instructions. The collagen mixture contained 8 parts of 3 mg/ml collagen solution to 1 part hydrogel media and 0.1 M sodium hydroxide (NaOH) to obtain a pH of about 7.4. To obtain a total of 10 parts, the remaining volume was adjusted with sterile distilled water. Four different hydrogel mixtures were tested. Fibrin gel served as the control hydrogel. The hydrogel mixtures tested were defined as the concentration of collagen gel per total hydrogel volume, with the remaining volume comprised of fibrin gel: Fibrin only; 0.7 mg/ml COL; 1.7 mg/ml COL; and 2.2 mg/ml COL. Using the pre-sterilized custom plates, 1.1 mL of the hydrogel mixture containing fibrin gel or a fibrin-collagen combination, depending on the experimental group, was added to the custom plate. The plate was then gently agitated by hand to ensure complete coverage of the plate. Next, 88 µL of thrombin was added, and the plate was again agitated. Finally, 354 µL of fibrinogen was added dropwise, followed by unidirectional swirling of the plate to ensure even distribution. The plates were left on a flat surface to set for 15 minutes at room temperature.

### Assembly of adventitia vessels

Adventitia vessels are defined as 6 rings stacked to form a vascular-like tubular structure. Adventitia vessels were created by stacking individual rings using our lab’s recently developed Ring Stacking Method^10^. Briefly, rings were carefully removed from the original ring formation plate with forceps and placed around the post of a custom-made taller plate with a central 1.2 cm long post to accommodate the length of the longer vessel. Six rings were stacked to form an adventitia vessel construct. Fibrin glue was applied between the individual rings to adhere them together; 40–60 µL of 100 U/mL thrombin was applied between each ring, followed by 40–60 µL of 20 mg/mL fibrinogen to form the fibrin glue. The fibrin glue sets in about 15 min, after which the vessel is ready for use.

### Mechanical tensile testing

Tensile testing was performed using an UStretch system with a 5 N load cell (CellScale, Waterloo, Ontario, Canada). Rings were stretched circumferentially until failure with custom-made hooks at a strain rate of 8 mm/min. The custom-made hooks were made using a layer of sandpaper that was glued to grip pads with plastic coated steel hooks. Rings were stretched on day 8 after seeding, that is, once rings had fully formed. Adventitia rings from both the collagen production stimulation groups and fibrin gel-collagen gel groups were tensile tested to determine which experimental parameters resulted in the highest tensile strength, or strongest, rings. Sample numbers for the cytokine stimulated groups were: AA first (n = 4), TGF-β first (n = 4), and AA + TGF-β (n = 4). Rings stimulated with AA only or TGF-β only did not maintain sufficient integrity to be tensile tested. Following determination of the optimal collagen stimulation parameters, as determined by the group with the highest tensile strength, stimulation parameters were kept constant while hydrogel composition was varied. Fibrin-collagen hydrogel ring groups were tensile tested with sample numbers of: Fibrin only (n = 20), 0.7 mg/ml COL (n = 17), 1.7 mg/ml COL (n = 13), and 2.2 mg/ml COL (n = 5). Sample size for 2.2 mg/ml COL rings was lower because rings of this hydrogel composition did not consistently form rings.

Out of the hydrogel groups, two groups exhibited the highest tensile test results: Fibrin only and 0.7 mg/ml COL rings. These hydrogel groups were further optimized by allowing 1 and 2 week culture times to promote further collagen maturation^20^ and increase strength. Ring stacks, i.e. adventitia vessels, were built of 6 rings and cultured for 1 or 2 weeks, then tensile tested circumferentially. Controls consisted of Fibrin only (n = 3) and 0.7 mg/ml COL (n = 3) vessels cultured for 1 day. Sample numbers were Fibrin only- 1 wk (n = 5); Fibrin only- 2 wk (n = 5); 0.7 mg/ml COL- 1 wk (n = 5); and 0.7 mg/ml COL- 2 wk (n = 5).

Furthermore, 2 wk cultured Fibrin only (n = 3) and 0.7 mg/ml COL vessels (n = 3) were longitudinally tensile tested to compare inter-ring strength. Ring stacks were stretched at a strain rate of 8 mm/min. The end of the ring stacks were adhered to sand paper using VetBond and were placed into tensile machine clamps.

### Hemodynamic testing

The hemodynamic properties were assessed by exposing the vessels to flow conditions and through burst pressure testing. Vessels were hemodynamically tested using a custom-made bioreactor with a peristaltic pump (WT600-2J, Longer Precision Pump Co. Ltd, Boonton, NJ), a glass media reservoir, polymer tubing, a custom-made bioreactor chamber and 3D printed vessel holders. Vessels were secured onto the 3D printed vessel holders using VetBond Tissue Adhesive (3 M, St. Paul, MN). Three hour flow duration tests were conducted on Fibrin only vessels (n = 3) and 0.7 mg/mL COL vessels (n = 3), both previously cultured for one week. Burst pressure testing was conducted on one week cultured Fibrin only (n = 3) and 0.7 mg/ml COL vessels (n = 3), and on a two week cultured 0.7 mg/mL COL vessel (n = 1). To determine burst pressure, vessels were subjected to increasing fluid pressure until failure.

### Histology

Hematoxylin and eosin (H&E); Masson’s trichrome; and picrosirius red stains were performed on adventitia ring groups. H&E provided cellular organization and extracellular matrix (ECM) structure information. Trichrome and picrosirius red stains highlighted collagen content with blue and red colored stains, respectively. A 3-ring vessel was stained with trichrome to visualize collagen content in the complete vessel.

### Polarized light microscopy

Picrosirius red stained samples of the optimized fibrin-collagen hydrogel groups was further analyzed under polarized light to ascertain collagen maturity and fiber thickness. Birefringence images were obtained through imaging with an Axiovert 200 microscope (Carl Zeiss, Oberkochen, Germany). Under polarized light, collagen maturity and fiber thickness are indicated by differences in color: immature/thin fibers appear green/yellow and mature/thick fibers appear orange/red.

### Scanning electron microscopy

Scanning electron microscopy (SEM) was performed using a JSM-6510LV system (Hitachi, Tokyo, Japan) in order to visualize continuity of the cell sheet-hydrogel aggregate during ring formation. Ring samples were sputter-coated in Argon gas with 20 nm of Au/Pd at 10 mA, 8 V, and 70 mTorr. Samples were then mounted into the SEM and micron-level images taken of the microarchitecture structure in the rings.

### Immunogenicity testing

To test immunogenicity, the optimized 0.7 mg/ml COL rings (n = 6) were cultured for 24 h at 37 °C submerged in human whole blood (SER-WB10ML-SDS, Zen-Bio, Inc., Research Triangle Park, NC). Following the 24 h period, rings were extracted, mounted in OCT, sectioned, and immunostained for CD45 to determine whether leukocytes had adhered to the engineered construct, possibly indicating potential to initiate the immune response.

### Thrombogenicity testing

To test thrombogenicity, four-ring 0.7 mg/ml COL vessels cultured for 2 weeks (n = 3) were incubated for 24 h at 37 °C in a solution of human platelet concentrate (SER-PC-SDS, Zen-Bio, Inc.). Samples were then removed from the platelet solution, mounted in OCT, and sectioned. Platelet adherence to the vessel was probed by immunostaining for CD41 platelet antibody. Evidence of adhesion of platelets to the vessel may indicate potential for the coagulation cascade to initiate leading to possible thrombi formation. In addition, lumen occlusion was assessed from the whole blood immersion immunogenicity experiments, as clotting factors are present in human whole blood.

### Statistics

Averages of tensile properties for the rings and vessels, and histology collagen quantification were reported as mean values ± standard deviation. One-way ANOVA was performed to compare the tensile testing results for the three groups of collagen production stimulation, the four groups of varying hydrogel composition rings, and for comparing the circumferential mechanical properties of Fibrin only and 0.7 mg/ml COL. A Tukey post-hoc test was performed to determine which groups were significantly different from one another. A two-tailed Student’s t test was used for the longitudinal mechanical properties, and collagen content and collagen maturity quantification analyses. Alpha value was set to 0.05. Statistics were calculated using SPSS (IBM, Armonk, New York). Detailed statistical information; including F values, *t* values and degree of freedom; can be found in Supplementary Tables 1–15.

## Results

### Optimized protocol for formation of adventitia rings and vessels

**Figure 1:**
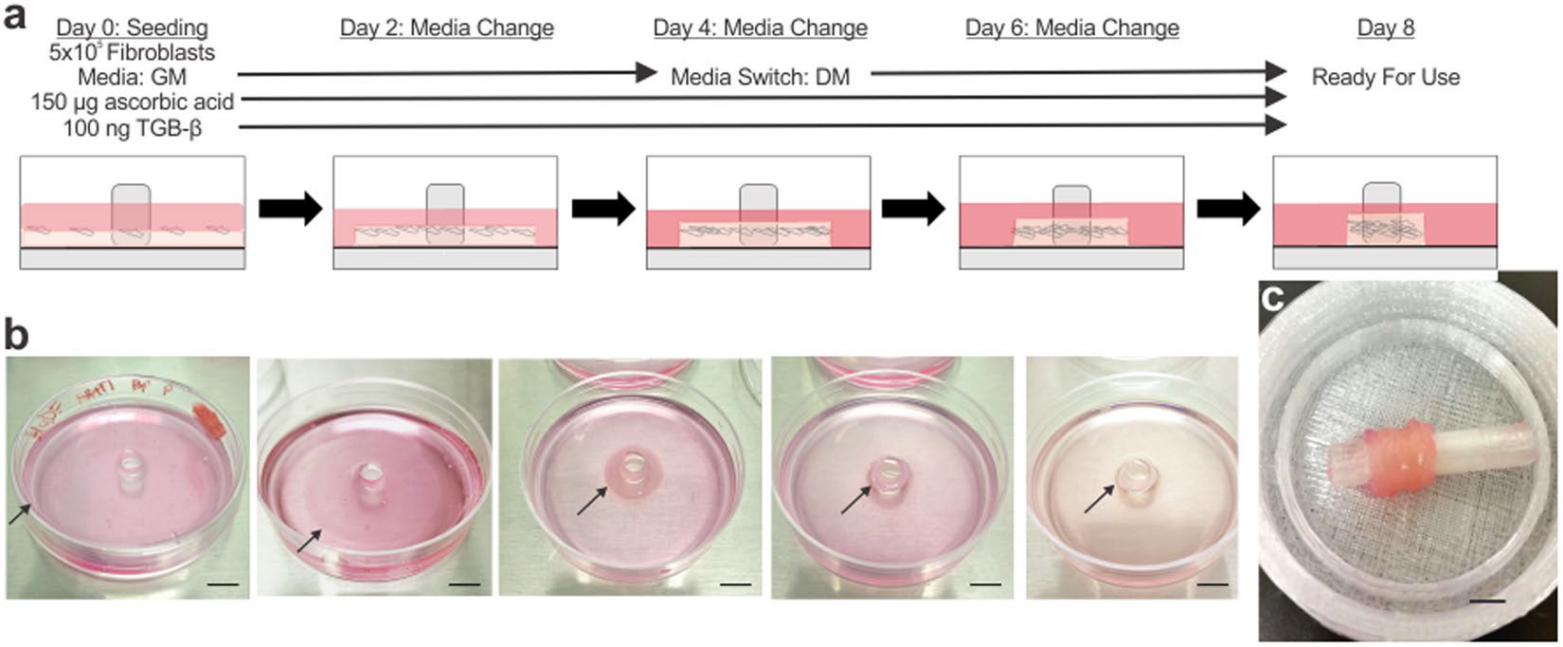
Optimized protocol for creating the engineered tunica adventitia. (**a**) Adventitia ring formation schematic showing the seeding density per 60 mm plate, media type and cytokine induction protocol. The hydrogel was added to custom-made plates with a bottom hydrophobic polymer coating and centrally placed posts. Human fibroblasts were then seeded in growth media (GM) and supplemented with ascorbic acid and TGF-β. On day 2, the media was changed with GM, and on days 4 and 6 media was switched to differentiation media (DM). Collagen production was enhanced by supplementing the media with both ascorbic acid and TGF-β at every media change. By day 8, the rings were ready for use. (**b**) Images of the adventitia rings self-organizing over the course of 8 days. Black arrows indicate the edge of cell sheet + hydrogel in the stages of the formation process from monolayer to ring (left to right). Scale bars = 1 cm. (**c**) Image of an adventitia vessel created by stacking 6 adventitia rings secured with biodegradable fibrin glue. Scale bar = 0.5 cm.

Self-assembled fibroblast monolayers formed into rings within 7 days (Fig. 1a). Optimal conditions utilized growth media during seeding followed by switching to differentiation media once the seeded cells had reached confluence. In addition, continuous cytokine stimulation using ascorbic acid and TGF-β throughout ring formation was required to form robust rings. The ring aggregation process from fibroblast monolayer to adventitia ring is shown in Fig. 1b. Adventitia rings were stacked and adhered using fibrin glue to create adventitia vessels (Fig. 1c).

**Figure 2:**
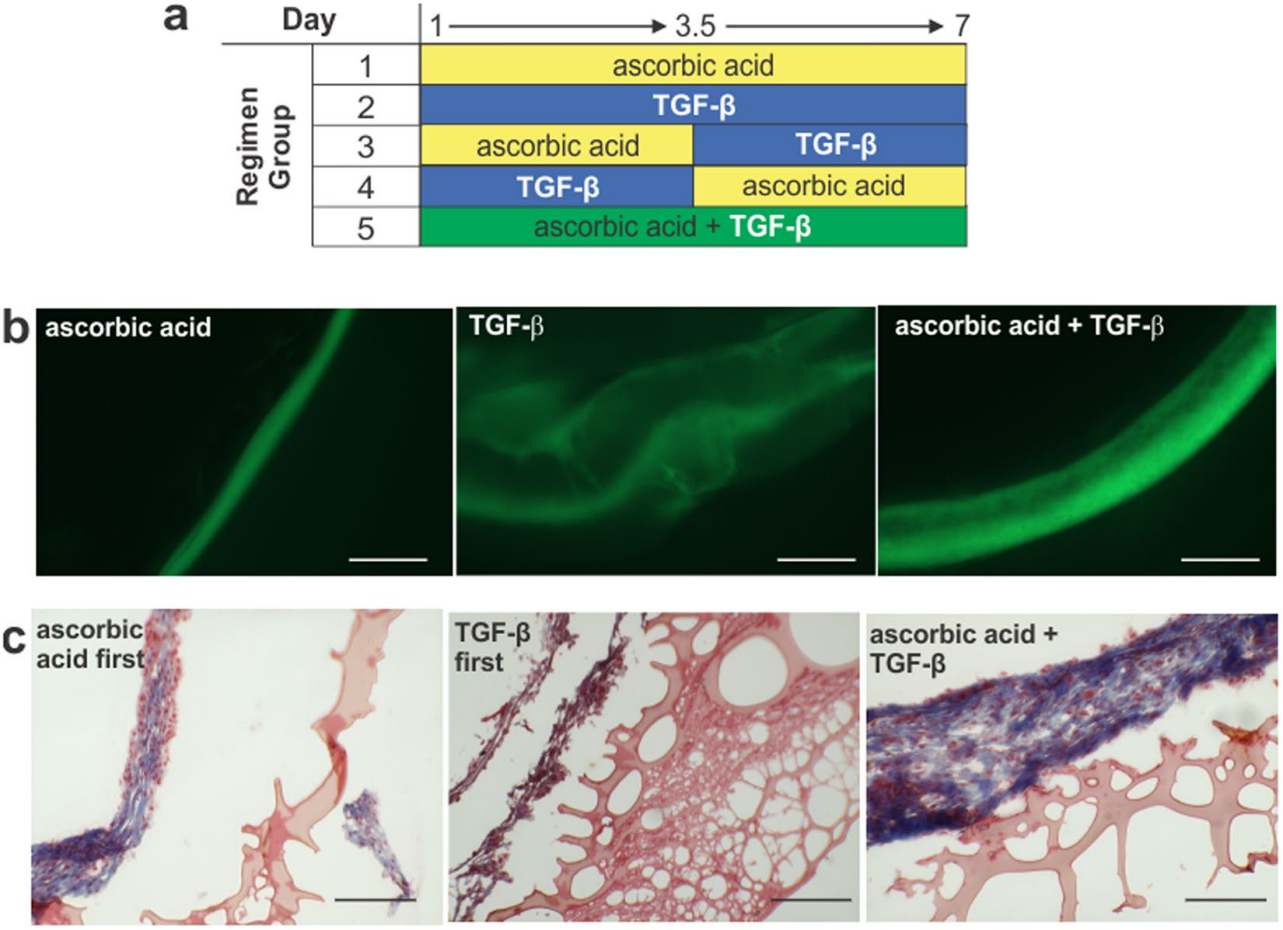
Cytokine regimen requires both ascorbic acid and TGF-β for adventitia ring formation. (**a**) Diagram of the five cytokine simulation regimens. (**b**) Images of fibroblast rings on day 8 under GFP light to visualize collagen auto-fluorescence with three different growth factor stimulations of groups: 1 (ascorbic acid only), 2 (TGF-β only) and 3 (combined ascorbic acid and TGF-β). Ascorbic acid only resulted in thin rings; TGF-β only resulted in loose rings. Combination rings resulted in thicker, robust rings. Scale bars = 500 µm. (**c**) Trichrome staining of adventitia ring cross-sections of groups: 3 (ascorbic acid first), 4 (TGF-β first) and 5 (ascorbic acid and TGF-β simultaneously). Combined stimulation throughout the ring formation resulted in the thickest, most robust rings with significant collagen content (blue/purplish stain). Scale bars = 200 µm.

Collagen production stimulations of ascorbic acid only, TGF-β only, and a combination of the two were tried (Fig. 2a). Collagen production from the initial stimulation groups was evaluated through visualizing the inherent auto-fluorescence attribute of collagen under GFP light excitation wavelength (Fig. 2b). Ring thickness varied among the groups: ascorbic acid only produced the thinnest rings at 157 ± 16.7 µm, which were too fragile to handle, and TGF-β only produced the thickest rings at 1831 ± 101 µm. Combining ascorbic acid and TGF-β produced the most robust rings at 386 ± 20.1 µm thick. Once it was determined that a combination of ascorbic acid and TGF-β was ideal for ring formation, the collagen production stimulation protocol was optimized.

Each collagen production stimulation factor was introduced individually, switching exposure halfway through the ring formation process, and then simultaneously added throughout ring formation (Fig. 2a). Samples from each group were stained with Trichrome to demarcate collagen in blue (Fig. 2c). Cytoplasm stains red, hence areas with collagen and cells appear purple. In the ascorbic acid first group, a small band of collagen around the cells can be seen. In the TGF-β first group, a thinner band of collagen is noted. In contrast, in the ascorbic acid + TGF-β group, a thick band of collagen and cells is observed. In corresponding tensile tests, tensile properties increased from the ascorbic acid first group to the TGF-β first group, with the combined cytokine group exhibiting the highest elasticity, ultimate tensile strength, and failure strength (Table 1). Tensile testing results between all groups and tensile properties were statistically significant (p < 0.01).

**Table 1:** Tensile properties of adventitia rings with different cytokine stimulation regimens.

Cytokine Group	Tensile Properties (kPa)		
	Elastic Modulus (E)	Ultimate Tensile Strength (UTS)	Failure Strength (FS)
ascorbic acid → TGF-β	81.7 ± 18.5^a^	15.2 ± 1.90^a^	4.43 ± 1.62^a^
TGF-β → ascorbic acid	170.6 ± 16.1^a^	40.0 ± 1.68^a^	36.5 ± 8.46^a^
ascorbic acid + TGF-β	257.6 ± 48.0^a^	57.8 ± 3.07^a^	56.9 ± 3.18^a^

^a^Denotes statistical significance between all groups (p < 0.01).

The final optimized protocol for creating adventitia rings begins with seeding of 5 × 10^5^ fibroblast cells in growth media in a 0.7 mg/ml COL hydrogel, switching to differentiation media at confluence, and the addition of both ascorbic acid and TGF-β from cell seeding until ring formation. Through this protocol, a 100% success rate of ring formation was achieved. The optimized protocol for creating adventitia vessels entails stacking the adventitia rings, adhering them together with fibrin glue, and allowing the vessels to culture for 1 week (based on the mechanical data).

### Mechanical properties of adventitia rings and vessels circumferentially tensile tested

Mechanical properties of adventitia rings were determined from stress-strain curves. Elastic moduli (E), ultimate tensile strengths (UTS), and failure strengths (FS) were obtained for cytokine stimulated groups of ascorbic acid first; TGF-β first; and ascorbic acid and TGF-β combined (Table 1). All 3 groups exhibited statistical significance between groups for all tensile properties of E, UTS and FS (p < 0.01). The ascorbic acid first group resulted in E, UTS and FS values of 81.7 ± 18.5 kPa, 15.2 ± 1.90 kPa, and 4.43 ± 1.62 kPa, respectively. The TGF-β first group resulted in E, UTS and FS values of 170.6 ± 16.1 kPa, 40.0 ± 1.68 kPa, and 36.5 ± 8.46 kPa, respectively. The combined collagen production stimulation group resulted in E, UTS and FS values of 257.6 ± 48.0 kPa, 57.8 ± 3.07 kPa, and 56.9 ± 3.18 kPa, respectively. The combined group displayed the highest elasticity and mechanical strength, thus it was determined that both ascorbic acid and TGF-β were needed for adventitia ring optimization.

**Figure 3:**
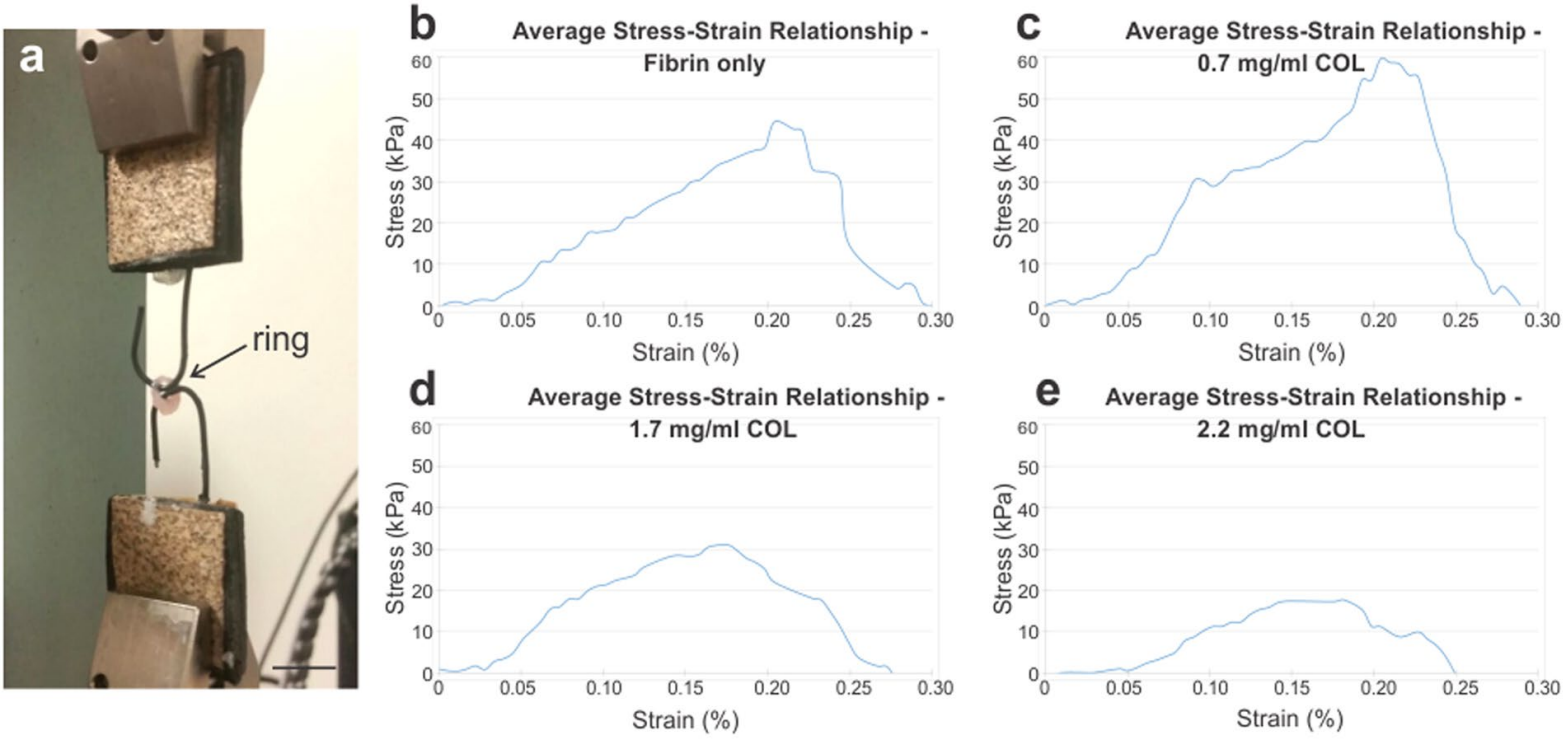
Adventitia rings with 0.7 mg/ml collagen exhibited highest tensile properties. (**a**) Tensile testing setup for the adventitia rings. Average stress-strain curves for (**b**) Fibrin only (n = 20); (**c**) 0.7 mg/ml COL (n = 17); (**d**) 1.7 mg/ml COL (n = 13); and (**e**) 2.2 mg/ml COL (n = 5) rings. The 0.7 mg/ml COL rings exhibited the highest tensile strength. Scale bar = 5 mm.

Next, hydrogel composition was optimized based on tensile test results (Fig. 3, Table 2). The ring tensile test setup is shown in Fig. 3a; average stress-strain curves for all hydrogel groups are shown in Fig. 3b–e. For the first group, Fibrin only, E, UTS and FS values were 265 ± 108 kPa, 7.1 ± 23.7 kPa, and 51.2 ± 24.9 kPa, respectively. For the 0.7 mg/ml COL group, E, UTS and FS values were 429 ± 134 kPa, 77.0 ± 18.1 kPa, and 53.4 ± 25.6 kPa, respectively. For the 1.7 mg/ml COL group, E, UTS and FS values were 461 ± 325 kPa, 39.6 ± 14.6 kPa, and 21.7 ± 14.8 kPa, respectively. Lastly, for the 2.2 mg/ml COL group, E, UTS and FS values were 203 ± 78.6 kPa, 21.6 ± 5.24 kPa, and 10.6 ± 5.26 kPa, respectively. The 1.7 mg/ml COL group had the highest elastic modulus, whereas the 0.7 mg/ml COL group had the highest ultimate tensile strength and failure strength. Hence, a 0.7 mg/ml collagen gel in a base fibrin gel was selected as the ideal hydrogel composition for the adventitia rings.

**Table 2:** Tensile properties of adventitia rings with differing hydrogel content.

Hydrogel Group	Tensile Properties (kPa)		
	Elastic Modulus (E)	Ultimate Tensile Strength (UTS)	Failure Strength (FS)
Fibrin only	265 ± 108	57.1 ± 23.7	51.2 ± 24.9
0.7 mg/ml COL	429 ± 134^a^	77.0 ± 18.1^a^	53.4 ± 25.6
1.7 mg/ml COL	461 ± 325^a^	39.6 ± 14.6^b^	21.7 ± 14.8^a,b^
2.2 mg/ml COL	203 ± 78.6	21.6 ± 5.24^a,b^	10.6 ± 5.26^a,b^

All groups were subjected to cytokine stimulation with TGF-β and ascorbic acid. Groups were defined in terms of percent of collagen gel (COL) in a base fibrin gel. ^a^Denotes statistical significance to fibrin only (p ≤ 0.01); ^b^Denotes statistical significance to 0.7 mg/ml COL (p ≤ 0.01).

**Figure 4:**
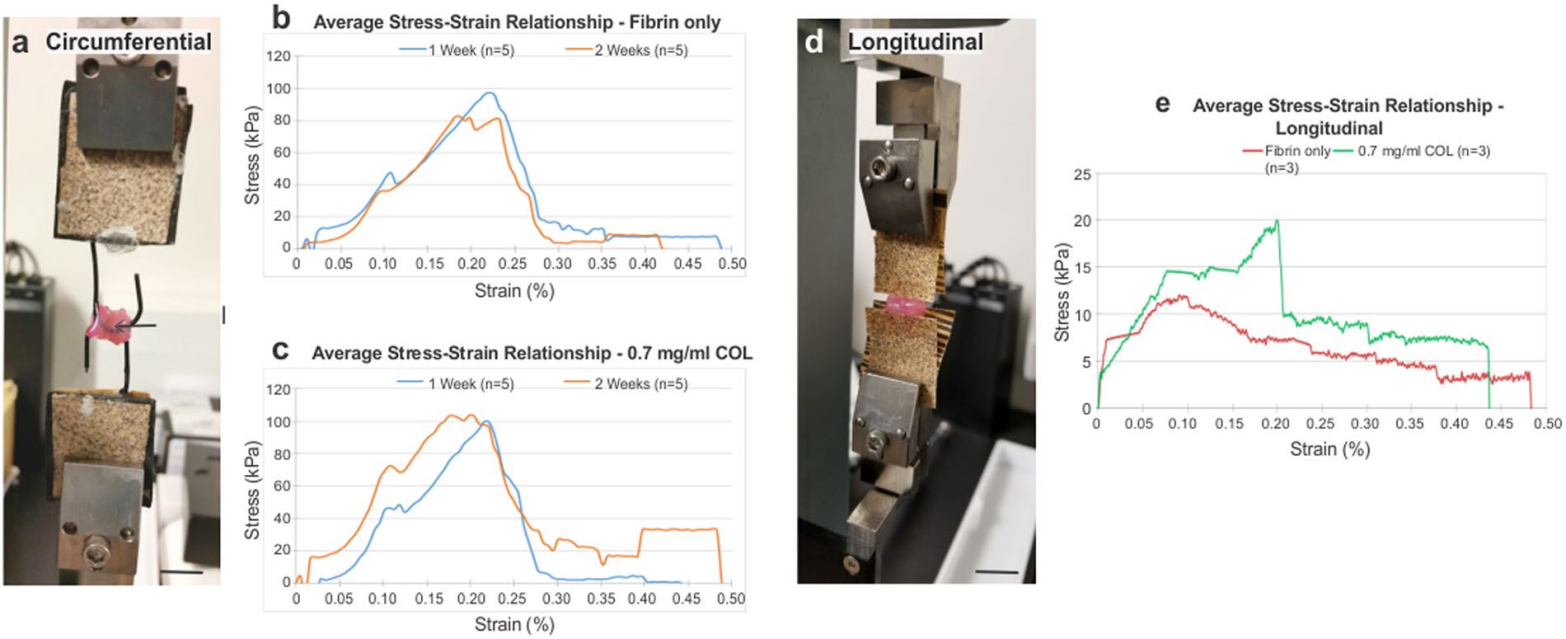
Adventitia vessels composed of 0.7 mg/ml collagen and cultured for 2 weeks exhibited highest ultimate tensile strength. Circumferential tensile testing (**a**), setup for the adventitia ring stacks (scale bar = 1 cm); (**b**), average stress-strain curves for Fibrin only vessels cultured for 1 week (n = 5) and 2 weeks (n = 5); and (**c**), average stress-strain curves for 0.7 mg/ml COL vessels cultured for 1 week (n = 5) and 2 weeks (n = 5). Ultimate tensile strength was highest for 0.7 mg/ml COL vessels cultured for 2 weeks, however, the difference in UTS between this group and the other three was non-significant. Longitudinal tensile testing (**d**), setup for the adventitia ring stacks with sandpaper grips on either end of the vessel length (scale bar = 1 cm); and (**e**), average stress-strain curves for 2 week cultured Fibrin only (n = 3) and 0.7 mg/ml COL vessels (n = 3). Average longitudinal UTS was similar for both groups, with a non-significant difference.

Culture time for the collagen-fibrin gel ring stacks was investigated, to determine if longer culture periods would result in increased vessel strength due to collagen maturation^20^. The two hydrogel groups tested were the Fibrin only and 0.7 mg/ml COL groups, and both were cultured for 1 and 2 weeks, and compared to vessels cultured for 1 day (Fig. 4, Table 3). The tensile testing setup for the vessels is shown in Fig. 4a; the average stress-strain curves are shown in Fig. 4b–c. The 0.7 mg/ml COL group cultured for 1 day and 2 weeks resulted in the highest values for E and UTS of 856 ± 99.2 kPa, and 119 ± 20.2 kPa, respectively, for the 1 day group, and 761 ± 215 kPa and 120 ± 23.6 kPa, respectively for the 2 week group. Failure strength was lowest in the 1 day groups. Differences in ultimate tensile strength were not significant. Significant differences in elastic moduli and failure strength are indicated in Table 3. The 0.7 mg/ml COL vessels had significant differences in elastic modulus between the 1 day, 1 week and 2 week groups. The Fibrin only vessels did not have significant differences in elastic modulus between its different culture period groups, though there were significant differences between the Fibrin only vessels and multiple 0.7 mg/ml COL groups. For failure strength, 1 day groups were significantly different from 1 week and 2 week groups for both the Fibrin only and 0.7 mg/ml COL vessels.

**Table 3:** Circumferential tensile properties of adventitia vessels cultured for 1 day, 1 week and 2 week periods.

Hydrogel vessel Group		Tensile Properties (kPa)		
		Elastic Modulus (E)	Ultimate Tensile Strength (UTS)	Failure Strength (FS)
1 d	Fibrin only (n = 3)	641 ± 114	108 ± 15.0	11.6 ± 5.24^c,d,e,f^
	0.7 mg/ml COL (n = 3)	856 ± 99.2^c,d^	119 ± 20.2	9.75 ± 4.45^c,d,e,f^
1 wk	Fibrin only (n = 5)	472 ± 94.1^b,f^	105 ± 12.4	49.9 ± 28.4^a^
	0.7 mg/ml COL (n = 5)	536 ± 119^b,f^	103 ± 28.9	58.9 ± 9.62^a^
2 wk	Fibrin only (n = 5)	576 ± 147^b,f^	103 ± 27.4	57.8 ± 18.0^a^
	0.7 mg/ml COL (n = 5)	771 ± 203^c,d,e^	120 ± 23.6	41.2 ± 6.41^a^

All groups used human fibroblasts for seeding of the 6-ring stack vessels and cytokine stimulation with TGF-β and ascorbic acid. ^a^Denotes statistical significance to fibrin only – 1 d (p ≤ 0.05). ^b^Denotes statistical significance to 0.7 mg/ml COL – 1 d (E: p ≤ 0.01; FS: p ≤ 0.05). ^c^Denotes statistical significance to fibrin only – 1 wk (p ≤ 0.05). ^d^Denotes statistical significance to 0.7 mg/ml COL – 1 wk (E: p ≤ 0.05; FS: p ≤ 0.001). ^e^Denotes statistical significance to fibrin only – 2 wk (E: p ≤ 0.05; FS: p ≤ 0.001). ^f^Denotes statistical significance to fibrin only – 2 wk (p ≤ 0.05).

### Mechanical properties of adventitia vessels longitudinally tensile tested

Adventitia vessels of Fibrin only (n = 3) and 0.7 mg/ml COL (n = 3) were cultured for 2 weeks and longitudinally stretched to failure (Fig. 4d,e; Table 4). Failure in both ring stack groups occurred between the rings. Longitudinal elastic modulus was higher, though not significantly, in the Fibrin only vessels compared to the 0.7 mg/ml COL vessels, with values of 537 ± 702 kPa and 249 ± 207 kPa, respectively. Both vessels exhibited similar material properties for UTS and FS, with values of 18.0 ± 8.47 kPa and 4.50 ± 1.38 kPa, respectively, for Fibrin only vessels, and values of 19.4 ± 11.5 kPa and 4.24 ± 3.23 kPa, respectively, for 0.7 mg/ml COL vessels. No statistical significance was found between the two groups for E, UTS, or FS.

**Table 4:** Longitudinal tensile properties of adventitia vessels cultured for 1 week.

Hydrogel Group	Tensile Properties (kPa)		
	Elastic Modulus (E)	Ultimate Tensile Strength (UTS)	Failure Strength (FS)
Fibrin only	537 ± 702	18.0 ± 8.47	4.50 ± 1.38
0.7 mg/ml COL	249 ± 207	19.4 ± 11.5	4.24 ± 3.23

All groups used human fibroblasts for seeding of the 6-ring stack vessels and cytokine stimulation with TGF-β and ascorbic acid.

### Hemodynamic properties

Three hour flow duration tests resulted in no evidence of leakage from one week cultured Fibrin only vessels (n = 3) and 0.7 mg/mL COL vessels (n = 3). One week cultured Fibrin only (n = 3) constructs exhibited a burst pressure of 44.4 ± 2.46 mmHg. Similarly, one week cultured 0.7 mg/ml COL vessels (n = 3) burst pressure was 45.6 ± 1.39 mmHg. This difference was not significant (p = 0.4). Burst pressure for a two week cultured 0.7 mg/mL COL vessel (n = 1) increased slightly to 51.7 mmHg. At pressures lower than burst pressure, vessels did not exhibit leakage along its length nor between rings. The perfusion system setup and a video of flow through a 0.7 mg/ml COL vessel is shown in Supplementary Fig. S1 and Video S1, respectively.

### Histological analysis and collagen content quantification

**Figure 5:**
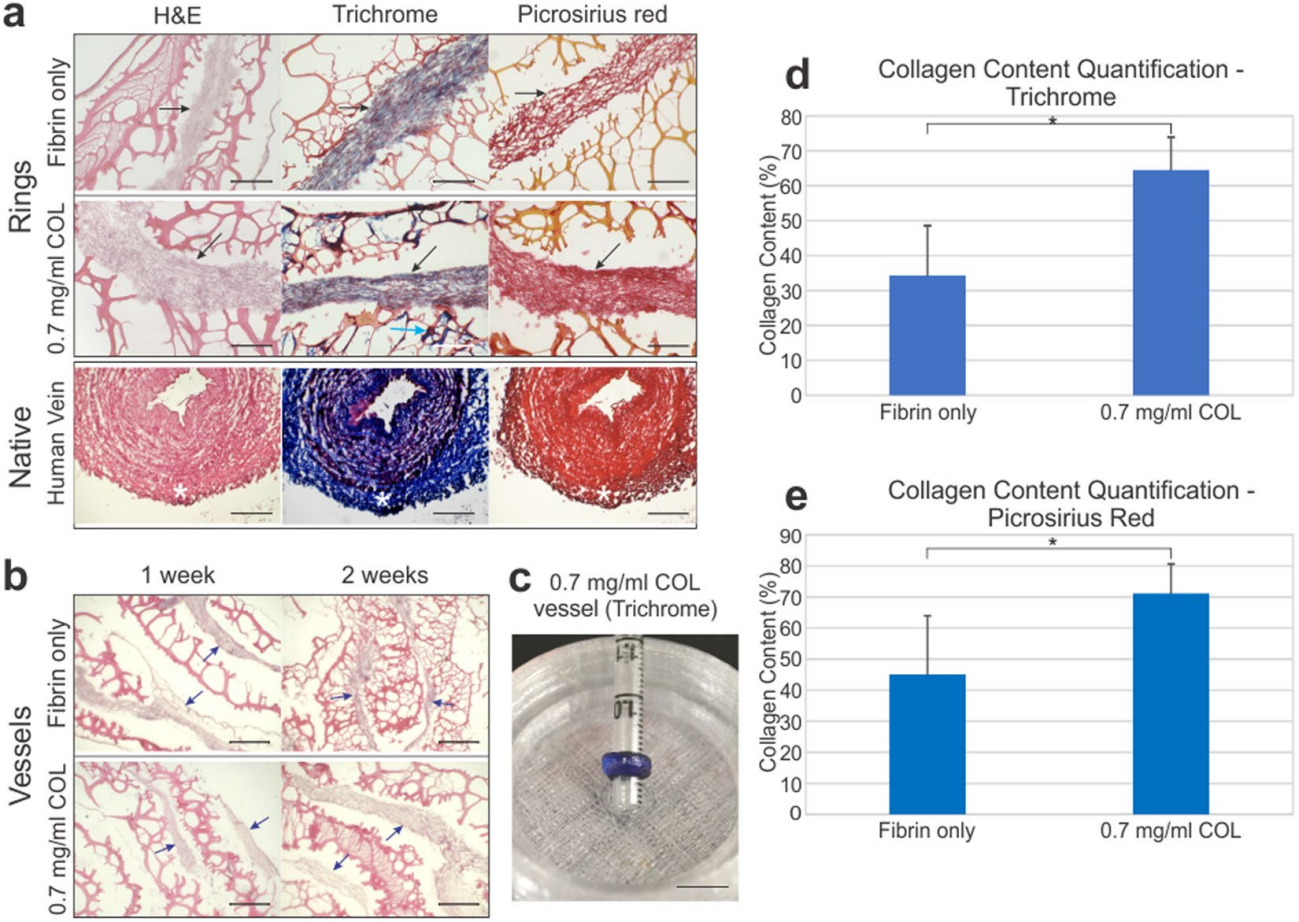
Histology of Fibrin only and 0.7 mg/ml collagen adventitia rings and vessels. (**a**) Collagen is present in H&E (pink), Masson’s Trichrome (blue/purple), and Picrosirius red (red) stains in ring cross-sections. Cell + collagen areas (black arrows) appears thicker and more dense in the 0.7 mg/ml COL rings compared to the Fibrin only rings. Additionally, collagen was present in the hydrogel on the periphery of the cellular region (Trichrome image, light blue arrow). Native human vein histological samples are shown for comparison; the adventitia layer is located on the outer edge of the native vein (white asterisk). The organization and density of the collagen in the engineered adventitia appears similar to that of the native vein. Engineered vessel scale bars = 200 µm. Native vein scale bars = 100 µm. (**b**) Longitudinal sections stained with H&E show the alternating organization of cells (blue arrows) and hydrogel along the ring stacked vessels in Fibrin only and 0.7 mg/ml COL vessels at 1 and 2 week culture times. Scale bars = 250 µm. (**c**) Masson’s Trichrome stain of a 3-ring stack of 0.7 mg/ml COL rings show the presence of (blue) collagen throughout the vessel. Scale bar = 1 cm. Collagen content quantification was performed using data from (**d**) Trichrome staining and (**e**) Picrosirius red staining. Collagen content was significantly higher in the 0.7 mg/ml COL rings compared to the Fibrin only rings (p < 0.0001). *Denotes statistical significance.

**Figure 6:**
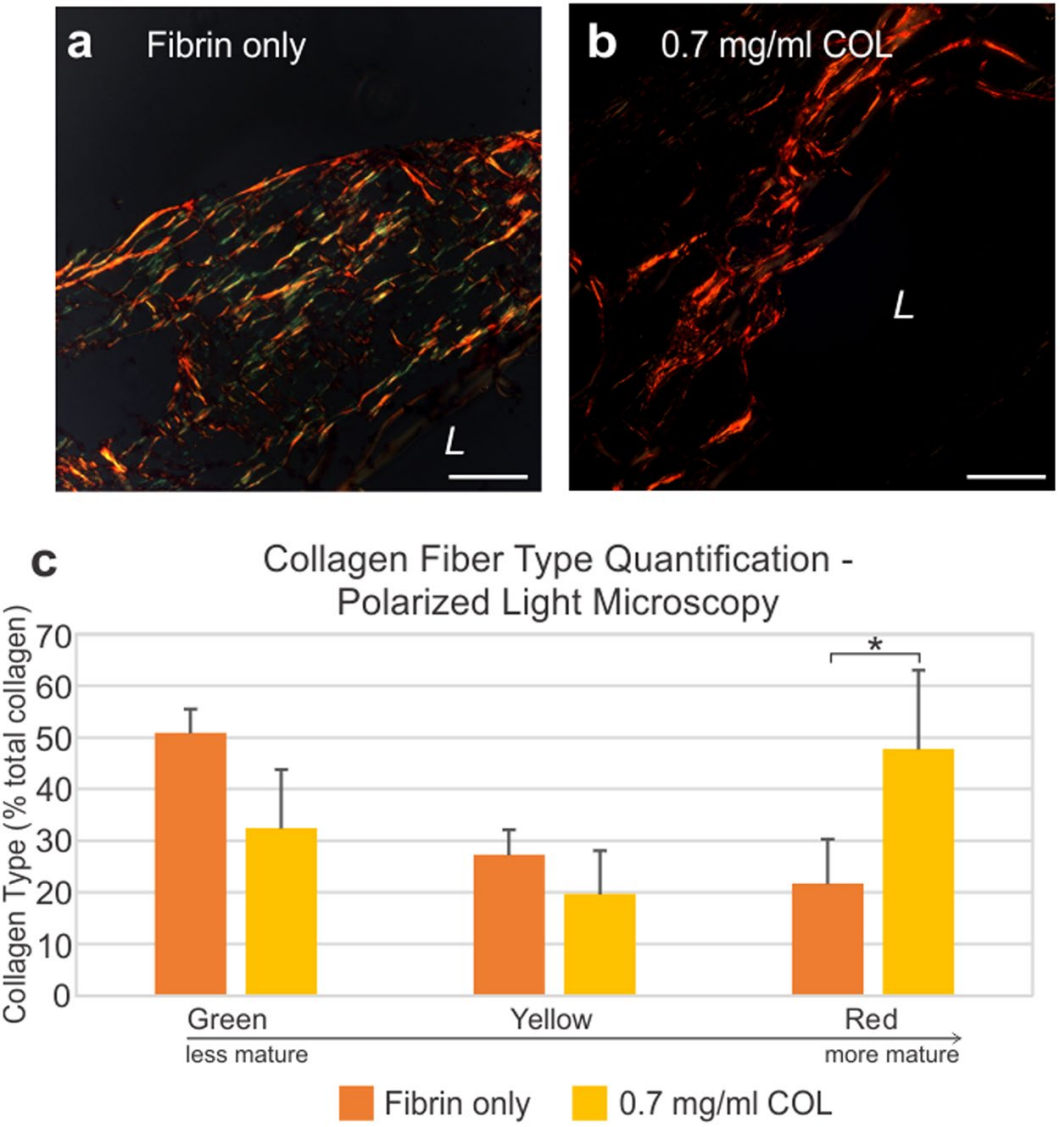
Polarized light images reveal a distinct difference in collagen maturity and fiber thickness between the Fibrin only and 0.7 mg/ml COL rings. (**a**) Rings with fibrin only displayed a mix of green, yellow and a few red fibers indicating a mixed composition of mostly immature collagen and some mature fibers. (**b**) Collagen in 0.7 mg/ml COL rings appeared as mature red fibers. The yellow and green colors signify the presence of thin fibers, and red indicates thick fibers. *L* = lumen side. Scale bars = 100 µm. (**c**) Collagen fiber type/maturity were measured using the polarized light images through green, yellow and red fiber quantification. Fibrin only rings exhibited more green fibers indicating less maturity, whereas 0.7 mg/ml COL rings displayed more red fibers indicating more maturity with a statistically significant difference (p ≤ 0.05).

The effects of the addition of the collagen gel and culture time on collagen content was evaluated through histology for H&E, Trichrome and Picrosirius red (Fig. 5), and through polarized light analysis (Fig. 6). Collagen appears pink in H&E, blue in Trichrome, and red in Picrosirius red. The 0.7 mg/ml COL rings consistently showed thicker and denser regions of cells and ECM (black arrows) compared to control Fibrin only rings (Fig. 5a). The web-like material on the periphery is the hydrogel. In the Fibrin only rings, collagen is present in the cellular region of the rings indicated by the pink stained nuclei in H&E. In the 0.7 mg/ml COL rings, blue marked collagen is present in the cellular region of the rings in addition to the hydrogel region of the ring (Fig. 5a, 0.7 mg/ml COL, Trichrome, green arrow). The Picrosirius red stained 0.7 mg/ml COL rings show a denser red stain in the cellular region compared to the red stain in Fibrin only rings. Similarly, in longitudinal sections showing multiple rings across a vessel stack, larger areas of cells and ECM are observed in the 0.7 mg/ml COL group compared to the Fibrin only group (Fig. 5b, blue arrows). Longitudinal sections along the vessels length show patterns of alternating cells/ECM (blue arrows) and hydrogel (Fig. 5b). The H&E stains of Fibrin only and 0.7 mg/ml COL vessel at 1 and 2 week time points show healthy cellularity. A trichrome stained 3-ring 0.7 mg/ml COL vessel showed consistent blue stain, indicating the presence of collagen throughout the tissue (Fig. 5c).

Collagen content as a percentage of the total area in the Fibrin only and 0.7 mg/ml COL rings was evaluated in the Trichrome and Picrosirius red images using ImageJ (NIH, Bethesda, MD) (Fig. 5d,e). Collagen content included all collagen present in the adventitia constructs, including collagen deposited by the fibroblasts and the collagen in the added collagen gel. In Trichrome, the percent area of collagen for Fibrin only and 0.7 mg/ml COL rings was 34.3 ± 14.3% and 64.5 ± 9.38%, respectively. In Picrosirius red, the percent area of collagen for Fibrin only and 0.7 mg/ml COL rings was 45.1 ± 18.9% and 71.2 ± 9.44%, respectively. The 0.7 mg/ml COL rings had a statistically significantly higher percent area of collagen as indicated by t test (p < 0.0001).

### Polarized light microscopy collagen fiber type quantification

Picrosirius red stained samples (Fig. 6a,b) were further analyzed using polarized light in order to determine maturity of collagen present within the tissue. Collagen color under polarized light indicate collagen maturity, with maturity increasing from green to yellow to red. The Fibrin only rings displayed green and yellow fibers signifying less maturity, whereas the 0.7 mg/ml COL rings were red indicating more maturity. Percent of green, yellow and red fibers per total collagen area for Fibrin only and 0.7 mg/ml COL rings were determined with ImageJ. Green, yellow, and red percent area of Fibrin only rings were 50.9 ± 4.57%, 27.3 ± 4.74% and 21.7 ± 8.55%, respectively. Green, yellow, and red percent area of 0.7 mg/ml COL rings were 32.5 ± 11.3%, 19.7 ± 8.30%, and 47.8 ± 15.2%, respectively (Fig. 6c). The percent area of red collagen fibers was statistically significantly greater in 0.7 mg/ml COL rings compared to Fibrin only rings (p ≤ 0.05). The percentage of green and yellow collagen fibers was greater in Fibrin only rings compared to 0.7 mg/ml COL rings, however it was not statistically significant.

### Adventitia ring microstructure

**Figure 7:**
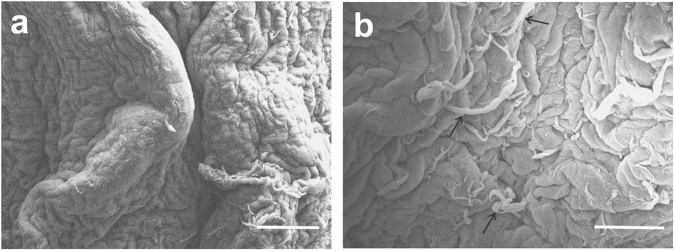
Scanning electron microscopy shows cell sheet continuity. (**a**) Cell sheet in the ring. (**b**) Collagen fibrils present within the cell sheet. Scale bars = 200 µm.

Scanning electron microscopy analysis showed continuity in the cell sheet that forms the ring structure (Fig. 7a). The cell monolayer appeared healthy, with no sign of rounded dead cells. Collagen fibrils can be seen interspersed throughout the fibroblast monolayer (Fig. 7b, black arrows).

### Immunogenicity

Treatment of the adventitia rings in whole human blood did not result in positive CD45 stain in the area of the ring tissue (see Supplementary Fig. S2). The whole human blood procured was unaltered and thus contained its normal white blood cell fraction. Attempts to mount and section samples of whole blood as a technical control were unsuccessful. However, whole blood leukocytes were found attached to the edge of a few sample slides located outside of the ring, remaining on the slide after PBS washes. These leukocytes did stain positive for CD45 (see Supplementary Fig. S2), showing that the antibody was functional and the staining protocol used was sound. No positive CD45 stain was found in the area of the ring itself.

### Thrombogenicity

**Figure 8:**
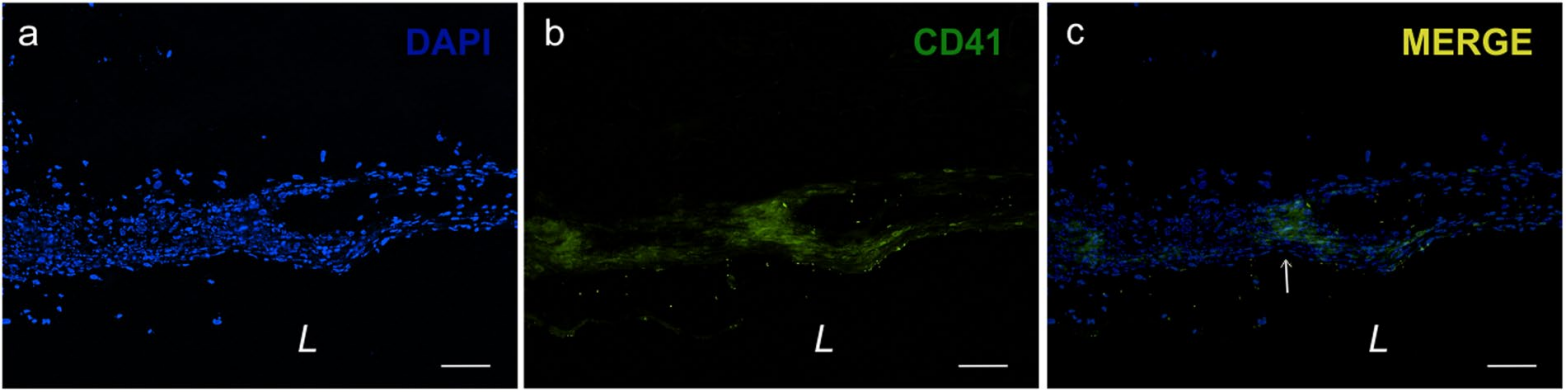
Very few areas of positive CD41 platelet stain indicate slight presence of adhered platelets to the adventitia rings. Inside the tissue rings, (**a**) fibroblast and platelet cell nuclei stained positive for DAPI, and (**b**) CD41 platelet stain was positive in very few 100 µm-sized areas near the edge of the rings. (**c**) Merged image shows co-localization of DAPI and CD41 stains (white arrow) indicating presence of platelets. *L* = lumen. Scale bars = 100 µm.

Few, small areas of platelet attachment was observed in the 0.7 mg/mL Col vessels as evidenced by CD41 platelet antibody stain (Fig. 8). Very sparse areas, about ~100 µm in size, of positive platelet stain were seen. Given that the thickness of the adventitia rings and vessels is about 0.5 cm, the few areas of attached platelets did not inundate the vessel. In addition, no clotting/occlusion was seen in 0.7 mg/mL rings submerged in whole blood for 24 h, further providing evidence supporting that there likely is a low probability that our vessels would occlude by thrombosis.

## Discussion

The tunica adventitia is favorable as a vascular graft because of its critical role in the structure of blood vessels. Vascular surgeons are able to repair vessels keeping very little tunica media and suturing mainly the adventitia during endarterectomy, suggesting that an engineered adventitia vessel could be a viable solution to off-the-shelf vessel repair.

The adventitia provides the primary mechanical strength to blood vessels. Hence, optimization of our engineered adventitia vessel protocol was based on tensile mechanics of the adventitia rings following each intervention. In the left anterior descending coronary artery, the circumferential tensile strength of the tunica adventitia is approximately 1430 ± 604 kPa^2,21^. The adventitia plays a key role in prevention of rupture under high pressures^2^. Our engineered adventitia vessels achieved a tensile strength of 120 ± 23.6 kPa. The cytokine stimulated, collagen gel adventitia vessel exhibits improved strength compared to our base technology, although the overall strength is less than native adventitia.

Our engineered adventitia could serve as an independent construct, or be combined with our previously developed engineered tunica media^13,14^. In anticipation of a bilayer vessel, modifications were made to the original ring formation protocol to allow for fabrication of 1 mm larger lumen size adventitia rings compared to our media rings. The 1 mm difference will allow for placement of the adventitia rings around the media rings in future studies. A 6 mm post size was used for the adventitia rings, compared to the 5 mm post size used to create media rings. In addition, a larger plate size (60 mm) was used for the adventitia rings compared to the media rings (35 mm). Each adventitia ring was approximately 1.5 mm in width, allowing for customization of the graft length.

Ascorbic acid and TGF-β are known to create stable extracellular matrix in fibroblast cultures and stimulate collagen production^15–19^. Ring formation times did not vary with stimulation type (i.e. ascorbic acid and/or TGF-β). Ascorbic acid is required for normal collagen production, by enabling synthesis of hydroxyproline and hydroxylysine^20^. Hydroxyproline stabilizes the collagen triple helix^22,23^, whereas hydroxylysine is needed in order for crosslinks to form between the collagen molecules^20^. TGF-β stimulates collagen I and III production in fibroblasts^24,25^. Ascorbic acid first rings had a thinner cellular region. The TGF-β first rings had thicker cellular regions with gaps within the tissue. When ascorbic acid and TGF-β were applied in conjunction, rings had thicker cellular regions with the highest amount of collagen (Fig. 2b). Combined ascorbic acid and TGF-β stimulation also resulted in the highest elastic modulus, ultimate tensile strength and failure strength. These results can be explained by the need for collagen production first prior to the need for the effects of hydroxyproline and hydroxylysine to occur. Hence, the conjunction of both cytokines maximizes the effects on collagen production and stabilization, allowing collagen to be stabilized and cross-linked as it is produced.

TGF-β is a common growth factor explored for vascular usage, including application of microbeads loaded with TGF-β into smooth muscle ring constructs^26^. The main focus in the Strobel *et al*. work was to determine a viable method for growth factor delivery through microbeads, however strength of their constructs did not change. Our present study offers a better outcome, with increased construct strength developed through not only TGF-β delivery through the easier, but just as effective, route of the media along with ascorbic acid and the addition of a collagen-fibrin hydrogel.

Hydrogel data did not include 100% COL rings because preliminary studies revealed that these rings were not able to consistently form. With a hydrogel composition of 100% collagen gel, ring formation had a success rate of about 40%. It was also difficult for rings to form in a 2.2 mg/ml collagen gel, with a success rate of 70%, whereas with the Fibrin only and 0.7 mg/ml COL hydrogels rings formed 100% of the time.

In terms of circumferential material properties, by incorporating collagen into the hydrogel, rings exhibited improved elasticity as indicated by the increase in elastic modulus (Table 2). The 1 day and 2 week 0.7 mg/ml COL vessels exhibited the highest elastic modulus and ultimate tensile strength values, with no significant difference between these two groups. Ultimate tensile strength did not change significantly between the different culture times, indicating that culture time did not have an effect on vessel ultimate tensile strength. In terms of elasticity, the significant decrease in elastic modulus in the 0.7 mg/ml COL vessels from day 1 to 1 week suggests that elastin remodeling may have occurred, causing a slight decrease in elasticity. Elastic modulus in the 2 week vessels was similar in the 1 week vessels, indicating that elastin remodeling had completed. Failure strength of both the Fibrin only and 0.7 mg/ml COL vessels was significantly higher at 1 and 2 weeks compared to the 1 day group. Fibrin gel, which is used to adhere the vessel rings together, has a strength in the range of 7.4–31.3 kPa^27^. The 1 day vessels exhibited a failure strength within the fibrin gel range (11.6 ± 5.24 kPa for fibrin only; 9.75 ± 4.45 kPa for 0.7 mg/ml COL), signifying that the dominant source of strength at that time was from the fibrin gel. Failure strength continued to increase over culture time, demonstrating that the rings had begun to integrate, and thus surpassing the strength of the fibrin gel. One day, 1 week and 2 week cultured 0.7 mg/ml COL vessels showed no difference in handleability.

Longitudinal UTS of our 2 week cultured Fibrin only and 0.7 mg/ml COL vessels for both vessels (18.0 ± 8.47 and 19.4 ± 11.5 kPa, respectively) was within the range of fibrin gel strength (7.4–31.3 kPa^27^). These results suggest that the ring-to-ring strength was maximized by the connecting fibrin gel. We are currently investigating methods to improve ring-to-ring adhesion, and thus strength, as described below in the burst pressure discussion.

Burst pressure values of our vessels are below that of native human saphenous vein (1680–2273 mmHg) and artery (2031–4225 mmHg)^11^. Our burst pressure of one week cultured vessels (~45 mmHg) and two week cultured vessels (51.7 mmHg) lays within the range for the burst pressure for fibrin gel (11.4–108.75 mmHg^27^), which preliminarily holds the rings together in our vessels. The fibrin gel degrades in about 4 weeks, during which time the cells will likely deposit additional ECM to further strengthen the vessel. We are currently exploring techniques to improve ring-to-ring adhesion and thus burst pressure, such as by increasing cell seeding number and applying additional hydrogel coatings, two techniques which have shown recent success in improving ring adhesion in our tunica media vessels constructed with vascular smooth muscle cells^13^. Positive results showed that our adventitia vessels did not leak at pressures up to burst pressure, showing there was no leakage between the rings in the vessel, thus exhibiting hemostasis.

Under polarized light, collagen fiber color indicate increasing fiber thickness from green to yellow to orange to red. Color is often mistaken to represent fiber type; rather, color indicates the maturity of the collagen fiber, with thick fibers indicating mature collagen (red) while thin fibers indicate immature collagen (green)^28^. The 0.7 mg/ml COL rings had higher collagen content (Fig. 5d,e) and collagen that appeared bright red under polarized light along the edges of the rings (Fig. 6b), which in transmittance images of Picrosirius red stains appears as hydrogel. Hence, the higher collagen content and collagen maturity in the 0.7 mg/ml COL rings can be attributed to the addition of collagen gel into the ring formation hydrogel. The Fibrin only rings under polarized light appeared primarily green and yellow with few red fibers, suggesting that these collagen fibers, predominately immature, were derived de novo from fibroblasts producing their own ECM.

Immunogenicity tests of whole human blood exposure showed negative results for our adventitia constructs. Although leukocyte adhesion to the rings was not observed as indicated by a negative CD45 stain, the human blood trial serves as a preliminary glimpse into the immunogenicity of our vessels. Similarly, the thrombogenicity tests are also a preliminary look at potential for thrombi formation. Whole blood *ex vivo* tests are often used in assessing vaccine immunogenicity^29^. In addition, platelets have been used *in vitro* in thrombosis models^30^. While our studies do provide information into the immunogenic and thrombogenic potential of our vessels, we are exploring animal model experiments to further test immunogenicity and thrombogenicity, as well as hemodynamics, in the near future. Regardless, these preliminary experiments provide some evidence to the immune response and thrombus formation potential of our vessels. An immune response is initiated by recognition of an antigen and clot formation is elicited by an injury site or foreign body/material. If our vessels are not recognized as an antigen, leukocytes would not adhere to our vessel thus resulting in a CD45 negative stain. The limited areas of positive CD41 platelet stain may suggest low probability of thrombosis formation. Further testing is planned for future experiments.

## Conclusions

Our 0.7 mg/ml COL vessels exhibited the overall best combination of ultimate tensile strength (the most important function for the adventitia), cellularity, collagen content and collagen fiber maturity. The base structure is provided by our unique ring formation and ring stacking technique. The fibrin gel in the constructs offer stability and the human fibroblasts provide a vital functional component able to respond to local cues and produce additional ECM to strengthen the rings. In an ongoing follow-up study, we have recently developed new methods allowing us to strengthen our adventitia vessels close to the native adventitia range. We anticipate completion of this study and releasing these novel results. Another ongoing study involves addressing the immune response by investigating cell source.

## Electronic supplementary material

Supplementary Information

Supplementary Video S1
