# Supplementary material for: Self-assembled Collagen-Fibrin Hydrogel Reinforces Tissue Engineered Adventitia Vessels Seeded with Human Fibroblasts
Source: Sci Rep. 2018 Feb 19;8:3294. doi: 10.1038/s41598-018-21681-7 (PMC5818609; doi:10.1038/s41598-018-21681-7)
Supplement: Supplementary file 1 — Supplementary Information [file 41598_2018_21681_MOESM1_ESM.pdf]

Self-assembled Collagen-Fibrin Hydrogel Reinforces Tissue Engineered Adventitia Vessels Seeded with Human Fibroblasts

Bijal Patel[1], Zhengfan Xu[1], Cameron B. Pinnock[1], Loay S. Kabbani[2], Mai T. Lam[1,3,*]

[1]Department of Biomedical Engineering, Wayne State University, Detroit, MI
[2]Department of Vascular Surgery, Henry Ford Health System, Detroit, MI
[3]Cardiovascular Research Institute, Wayne State University, Detroit, MI

Addresses:

Bijal Patel, M.S.E.
Wayne State University
818 W. Hancock Street
Detroit, MI 48201 USA
bijal.patel3@wayne.edu

Zhengfan Xu, M.D.
Wayne State University
818 W. Hancock Street
Detroit, MI 48201 USA
fc8031@wayne.edu

Cameron B. Pinnock, M.S.E.
Wayne State University
818 W. Hancock Street
Detroit, MI 48201 USA
dw7722@wayne.edu

Loay Kabbani, M.D.
2799 W Grand Blvd
Detroit, MI 48202 USA
LKABBAN1@hfhs.org

Corresponding author:

Mai T. Lam, Ph.D.
Wayne State University
818 W. Hancock Street
Detroit, MI 48201 USA
mtlam@wayne.edu
Phone: (313) 577-0118
Fax: (313) 577-8333

Supplementary Figure S1 | Vessel undergoing hemodynamic testing in the custom-built perfusion system. Scale bar = 10 cm.

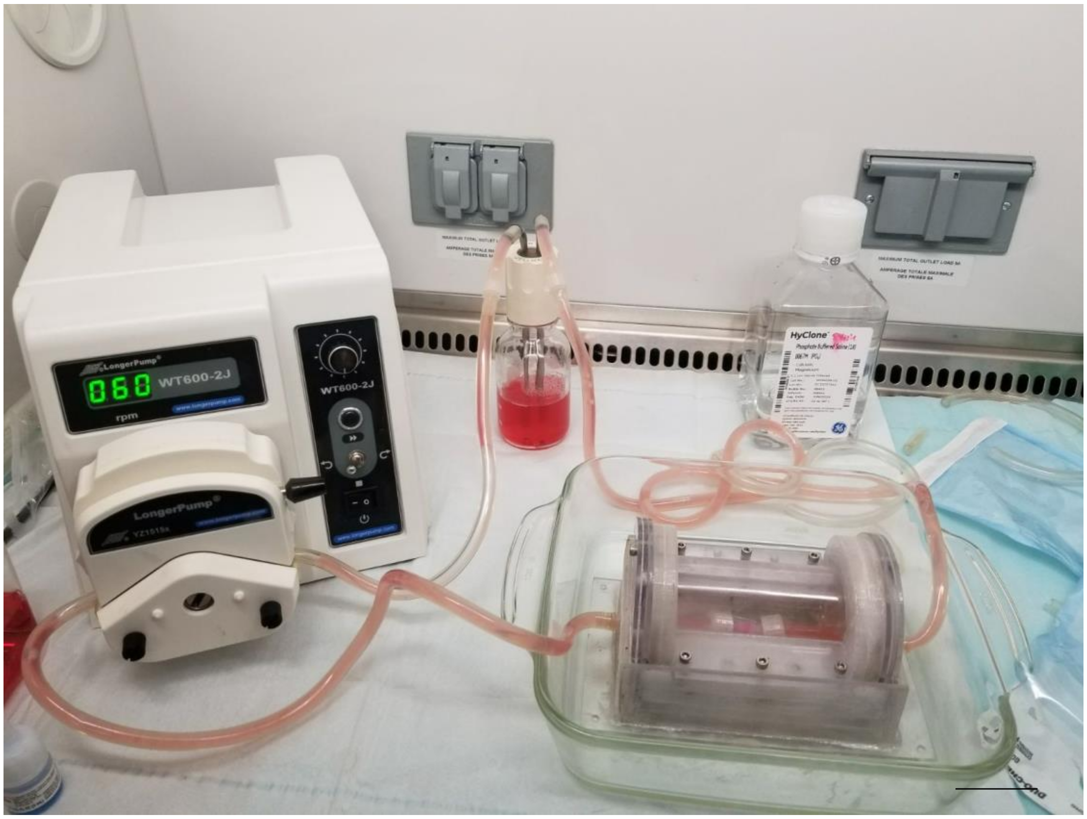

Supplementary Figure S2 | Negative CD45 stain indicates no presence of adhered leukocytes to the adventitia rings. Inside the tissue rings, **a,** fibroblast cells stained positive for DAPI, and **b,** CD45 leukocyte stain was negative. Outside the tissue ring, **c,** DAPI and **d,** CD45 stains were positive for the leukocytes. *L* = lumen. Scale bars = 100 µm.

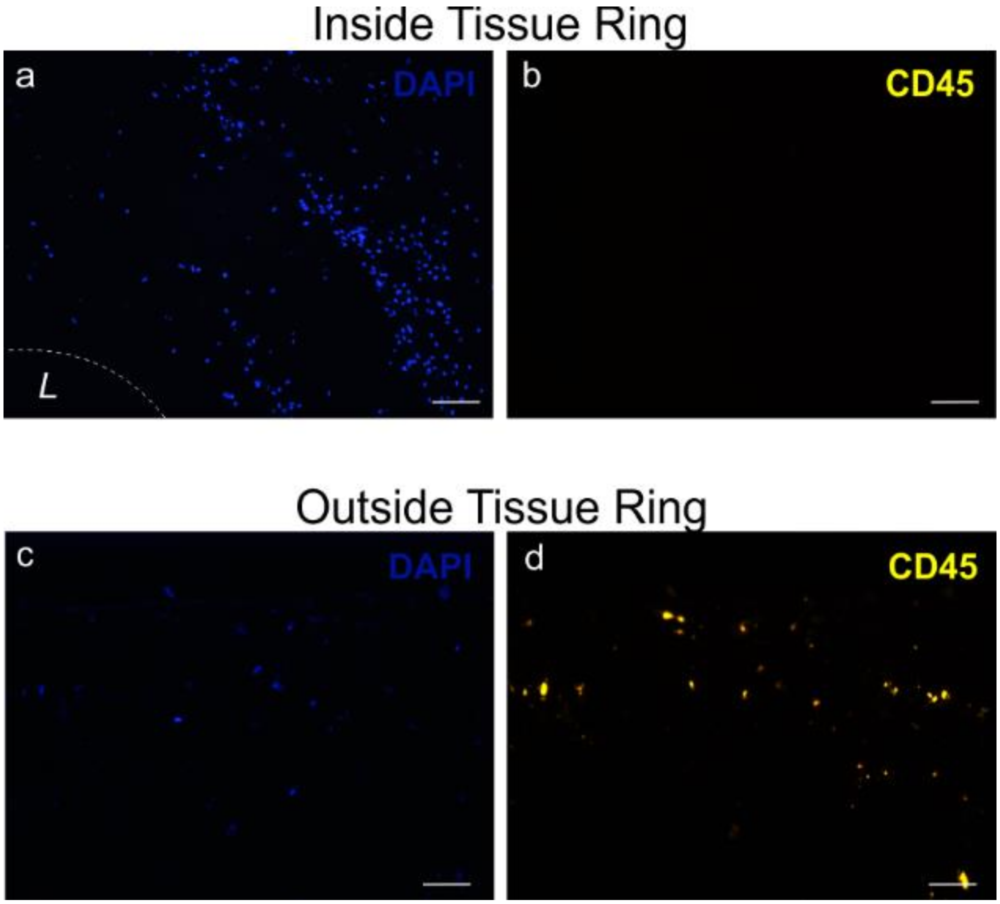

Supplementary Tables 1-4 | Statistics for tensile properties of adventitia rings with different cytokine stimulation regimens.

Cytokine Group	Tensile Properties ANOVA		
	Elastic Modulus (E)	Ultimate Tensile Strength (UTS)	Failure Strength (FS)
F Value	31.9	348	99.7
DF between groups	2	2	2
DF within groups	9	9	9
P Value	8.30×10^{-5}	2.99×10^{-9}	7.22×10^{-7}

Cytokine Group	Tensile Properties ANOVA P Values E		
	ascorbic acid → TGF-β	TGF-β → ascorbic acid	ascorbic acid + TGF-β
ascorbic acid → TGF-β		7.49×10^{-3}	6.00×10^{-5}
TGF-β → ascorbic acid	7.49×10^{-3}		8.49×10^{-3}
ascorbic acid + TGF-β	6.00×10^{-5}	8.49×10^{-3}	

Cytokine Group	Tensile Properties ANOVA P Values UTS		
	ascorbic acid → TGF-β	TGF-β → ascorbic acid	ascorbic acid + TGF-β
ascorbic acid → TGF-β		2.78×10^{-7}	2.66×10^{-8}
TGF-β → ascorbic acid	2.78×10^{-7}		4.00×10^{-6}
ascorbic acid + TGF-β	2.66×10^{-8}	4.00×10^{-6}	

Cytokine Group	Tensile Properties ANOVA P Values FS		
	ascorbic acid → TGF-β	TGF-β → ascorbic acid	ascorbic acid + TGF-β
ascorbic acid → TGF-β		3.40×10^{-5}	5.66×10^{-7}
TGF-β → ascorbic acid	3.40×10^{-5}		1.06×10^{-3}
ascorbic acid + TGF-β	5.66×10^{-7}	1.06×10^{-3}	

Supplementary Tables 5-8 | Statistics for tensile properties of adventitia rings with varying hydrogel content. Groups were defined in terms of collagen (COL) gel concentration in a base fibrin gel.

Hydrogel Ring Group	Tensile Properties ANOVA		
	Elastic Modulus (E)	Ultimate Tensile Strength (UTS)	Failure Strength (FS)
F Value	4.68	13.8	8.43
DF between groups	3	3	3
DF within groups	49	49	49
P Value	5.94×10^{-3}	1.00×10^{-6}	1.28×10^{-4}

Hydrogel Ring Group	Tensile Properties ANOVA P Values E			
	Fibrin only COL	0.7 mg/ml COL	1.7 mg/ml COL	2.2 mg/ml COL
Fibrin only COL		0.0479	0.0276	0.928
0.7 mg/ml COL	0.0479		0.966	0.140
1.7 mg/ml COL	0.0276	0.966		0.0874
2.2 mg/ml COL	0.928	0.140	0.0874	

Hydrogel Ring Group	Tensile Properties ANOVA P Values UTS			
	Fibrin only COL	0.7 mg/ml COL	1.7 mg/ml COL	2.2 mg/ml COL
Fibrin only COL		0.0150	0.0766	8.26×10^{-3}
0.7 mg/ml COL	0.0150		2.80×10^{-5}	2.60×10^{-5}
1.7 mg/ml COL	0.0766	2.80×10^{-5}		0.382
2.2 mg/ml COL	8.26×10^{-3}	2.60×10^{-5}	0.382	

Hydrogel Ring Group	Tensile Properties ANOVA P Values FS			
	Fibrin only COL	0.7 mg/ml COL	1.7 mg/ml COL	2.2 mg/ml COL
Fibrin only COL		0.991	4.05×10^{-3}	9.64×10^{-3}
0.7 mg/ml COL	0.991		2.64×10^{-3}	6.74×10^{-3}
1.7 mg/ml COL	4.05×10^{-3}	2.64×10^{-3}		0.830
2.2 mg/ml COL	9.64×10^{-3}	6.74×10^{-3}	0.830	

Supplementary Tables 9-12 | Statistics for circumferential tensile properties of adventitia vessels cultured for 1 day, 1 week and 2 week periods.

Hydrogel vessel Group	Tensile Properties ANOVA		
	Elastic Modulus (E)	Ultimate Tensile Strength (UTS)	Failure Strength (FS)
F Value	4.38	0.505	6.18
DF between groups	5	5	5
DF within groups	18	18	18
P Value	8.78×10^{-3}	0.768	1.68×10^{-3}

Hydrogel vessel Group	Tensile Properties ANOVA P Values E					
	Fibrin only COL – 1 day	0.7 mg/ml COL – 1 day	Fibrin only COL – 1 wk	0.7 mg/ml COL – 1 wk	Fibrin only COL – 2 wk	0.7 mg/ml COL – 2 wk
Fibrin only COL – 1 day		0.0685	0.108	0.330	0.528	0.225
0.7 mg/ml COL – 1 day	0.0685		0.00115	0.00657	0.116	0.425
Fibrin only COL – 1 wk	0.108	0.00115		0.492	0.242	0.00424
0.7 mg/ml COL – 1 wk	0.330	0.00657	0.492		0.666	0.0254
Fibrin only COL – 2 wk	0.528	0.0116	0.242	0.666		0.0471
0.7 mg/ml COL – 2 wk	0.225	0.425	0.0424	0.0254	0.0471	

Hydrogel vessel Group	Tensile Properties ANOVA P Values UTS					
	Fibrin only COL – 1 day	0.7 mg/ml COL – 1 day	Fibrin only COL – 1 wk	0.7 mg/ml COL – 1 wk	Fibrin only COL – 2 wk	0.7 mg/ml COL – 2 wk
Fibrin only COL – 1 day		0.547	0.840	0.775	0.755	0.476
0.7 mg/ml COL – 1 day	0.547		0.384	0.356	0.329	0.944
Fibrin only COL – 1 wk	0.840	0.384		0.916	0.899	0.307
0.7 mg/ml COL – 1 wk	0.775	0.356	0.916		0.989	0.286
Fibrin only COL – 2 wk	0.755	0.329	0.899	0.989		0.256
0.7 mg/ml COL – 2 wk	0.476	0.944	0.307	0.286	0.256	

Hydrogel vessel Group	Tensile Properties ANOVA P Values FS					
	Fibrin only COL – 1 day	0.7 mg/ml COL – 1 day	Fibrin only COL – 1 wk	0.7 mg/ml COL – 1 wk	Fibrin only COL – 2 wk	0.7 mg/ml COL – 2 wk
Fibrin only COL – 1 day		0.893	0.00563	0.00160	0.00134	0.0323
0.7 mg/ml COL – 1 day	0.893		0.00403	0.00116	0.000954	0.0239
Fibrin only COL – 1 wk	0.00563	0.00403		0.432	0.464	0.446
0.7 mg/ml COL – 1 wk	0.00160	0.00116	0.432		0.922	0.150
Fibrin only COL – 2 wk	0.00134	0.000954	0.464	0.922		0.155
0.7 mg/ml COL – 2 wk	0.0323	0.0239	0.446	0.150	0.155	

Supplementary Table 13 | Longitudinal tensile properties of adventitia vessels cultured for a 2 week period

Hydrogel Ring Group	Longitudinal Tensile Properties Two Tailed T test		
	Elastic Modulus (E)	Ultimate Tensile Strength (UTS)	Failure Strength (FS)
DF	4	4	4
t	0.679	-0.167	0.130
P Value	0.057	0.608	0.132

Supplementary Table 14 | Statistics for collagen content quantification for fibrin only collagen and 0.7 mg/ml collagen rings.

Hydrogel Ring Group	Collagen Content (% area) Two Tailed T test	
	Trichrome	Picrosirius red
DF	12	107
t	-4.79	-5.53
P Value	4.38×10^{-4}	2.29×10^{-7}

Supplementary Table 15 | Collagen type quantification for fibrin only collagen and 0.7 mg/ml collagen rings.

Hydrogel Ring Group	Collagen Type (% of total collagen) - Two tailed t test		
	Green (less mature)	Yellow	Red (more mature)
DF	17	17	17
t	1.59	1.31	-2.11
P Value	0.130	0.209	0.0498
